# Effect of a Computer-Based Decision Support Intervention on Autism Spectrum Disorder Screening in Pediatric Primary Care Clinics: A Cluster Randomized Clinical Trial

**DOI:** 10.1001/jamanetworkopen.2019.17676

**Published:** 2019-12-18

**Authors:** Stephen M. Downs, Nerissa S. Bauer, Chandan Saha, Susan Ofner, Aaron E. Carroll

**Affiliations:** 1 Division of Children’s Health Services Research, Department of Pediatrics, Indiana University School of Medicine, Indianapolis; 2 Regenstrief Institute Inc, Indianapolis, Indiana; 3 Axon Health Associates LLC, Indianapolis, Indiana; 4 Department of Biostatistics, Indiana University School of Medicine, Indianapolis; 5 Division of Pediatric and Adolescent Comparative Effectiveness Research, Department of Pediatrics, Indiana University School of Medicine, Indianapolis

## Abstract

**Importance:**

Universal early screening for autism spectrum disorder (ASD) is recommended but not routinely performed.

**Objective:**

To determine whether computer-automated screening and clinical decision support can improve ASD screening rates in pediatric primary care practices.

**Design, Setting, and Participants:**

This cluster randomized clinical trial, conducted between November 16, 2010, and November 21, 2012, compared ASD screening rates among a random sample of 274 children aged 18 to 24 months in urban pediatric clinics of an inner-city county hospital system with or without an ASD screening module built into an existing decision support software system. Statistical analyses were conducted from February 6, 2017, to June 1, 2018.

**Interventions:**

Four clinics were matched in pairs based on patient volume and race/ethnicity, then randomized within pairs. Decision support with the Child Health Improvement Through Computer Automation system (CHICA) was integrated with workflow and with the electronic health record in intervention clinics.

**Main Outcomes and Measures:**

The main outcome was screening rates among children aged 18 to 24 months. Because the intervention was discontinued among children aged 18 months at the request of the participating clinics, only results for those aged 24 months were collected and analyzed. Rates of positive screening results, clinicians’ response rates to screening results in the computer system, and new cases of ASD identified were also measured. Main results were controlled for race/ethnicity and intracluster correlation.

**Results:**

Two clinics were randomized to receive the intervention, and 2 served as controls. Records from 274 children (101 girls, 162 boys, and 11 missing information on sex; age range, 23-30 months) were reviewed (138 in the intervention clinics and 136 in the control clinics). Of 263 children, 242 (92.0%) were enrolled in Medicaid, 138 (52.5%) were African American, and 96 (36.5%) were Hispanic. Screening rates in the intervention clinics increased from 0% (95% CI, 0%-5.5%) at baseline to 68.4% (13 of 19) (95% CI, 43.4%-87.4%) in 6 months and to 100% (18 of 18) (95% CI, 81.5%-100%) in 24 months. Control clinics had no significant increase in screening rates (baseline, 7 of 64 children [10.9%]; 6-24 months after the intervention, 11 of 72 children [15.3%]; *P* = .46). Screening results were positive for 265 of 980 children (27.0%) screened by CHICA during the study period. Among the 265 patients with positive screening results, physicians indicated any response in CHICA in 151 (57.0%). Two children in the intervention group received a new diagnosis of ASD within the time frame of the study.

**Conclusions and Relevance:**

The findings suggest that computer automation, when integrated with clinical workflow and the electronic health record, increases screening of children for ASD, but follow-up by physicians is still flawed. Automation of the subsequent workup is still needed.

**Trial Registration:**

ClinicalTrials.gov identifier: NCT01612897

## Key Points

**Question:**

Can computer automation coupled with decision support increase timely screening for autism spectrum disorders in primary care practice?

**Findings:**

This cluster randomized clinical trial found that computer-based screening and decision support embedded into the routine workflow in primary care increased rates of screening from 0% to 100%, but physicians responded to approximately half of positive screening results.

**Meaning:**

Automating the screening process can ensure that screening takes place, but follow-through of the results is vulnerable to human error.

## Teaser

This randomized clinical trial examines whether computer-automated screening and clinical decision support can improve autism spectrum disorder screening rates in a pediatric primary care practice.

## Introduction

Autism spectrum disorder (ASD) represents a range of disabilities in speech, social interaction, and intellect, featuring repetitive stereotyped movements or behaviors, ranging from mild to severe.^1^ The prevalence of ASD in the United States has increased during recent decades.^2^ The Centers for Disease Control and Prevention estimates the prevalence of ASDs today to be 1 in 59 individuals.^2^

Therapies for ASD, notably applied behavioral analysis, have been shown to be effective.^3,4,5,6,7^ Controlled studies have demonstrated that applied behavioral analysis can result in significant increases in IQ,^5,6,7^ even into the normal range,^7^ with improved likelihood of mainstreaming in school.^8^ One study suggested that therapy with applied behavioral analysis could save more than $200 000 per child.^9^

However, the effectiveness of applied behavioral analysis depends on early initiation. There is an association between how early children begin therapy and the benefit they experienced.^3,4,6,10^ The likelihood that a child will benefit from applied behavioral analysis decreases with age; however, many children with ASD receive the diagnosis at an older age. Autism spectrum disorder can be diagnosed as early as age 16 months,^11,12^ yet the mean age at diagnosis in the United States is 4.5 years.^13^

For these reasons, the American Academy of Pediatrics (AAP) has recommended that primary care physicians caring for toddlers routinely screen for ASD at the 18-month visit and 24-month visit.^14^ Several screening instruments are available, but the most widely used is the 23-item Modified Checklist for Autism in Toddlers with Follow-up (M-CHAT-F), since revised to the 20-item M-CHAT-R/F. This instrument has a positive predictive value of 50% and can be administered in less than 10 minutes.^12^ Moreover, the M-CHAT-F is free and easy to download from the internet.^15^ Despite this, fewer than half of primary care physicians routinely screen toddlers for ASD.^13^

Over the last 14 years, we have developed and expanded a computer-based clinical decision support system called Child Health Improvement Through Computer Automation (CHICA), which has been shown to improve guideline-based care for a range of clinical topics.^16,17,18,19,20,21,22,23^ The purpose of this study was to evaluate the use of CHICA to improve ASD screening and follow-up in a randomized clinical trial among a group of community health centers.

## Methods

This cluster randomized clinical trial was performed between November 16, 2010, and November 21, 2012, among 4 pediatric primary care clinics that use CHICA. Four clinics were chosen from the 5 that use CHICA, and they were matched in pairs based on size and racial/ethnic distribution among patients. Within the pairs, the clinics were randomized to use CHICA with built-in ASD decision support or to continue using CHICA without ASD decision support. Randomization by cluster precludes exact matching of patients but avoids contamination within clinics. Randomizing by patient results in the system providing different advice for similar patients; physicians can become confused and irritated at this outcome. Moreover, physicians may use materials intended for intervention patients on their control patients. Thus, despite the liabilities of randomizing by clinic, we chose this approach.

**Figure 1.**
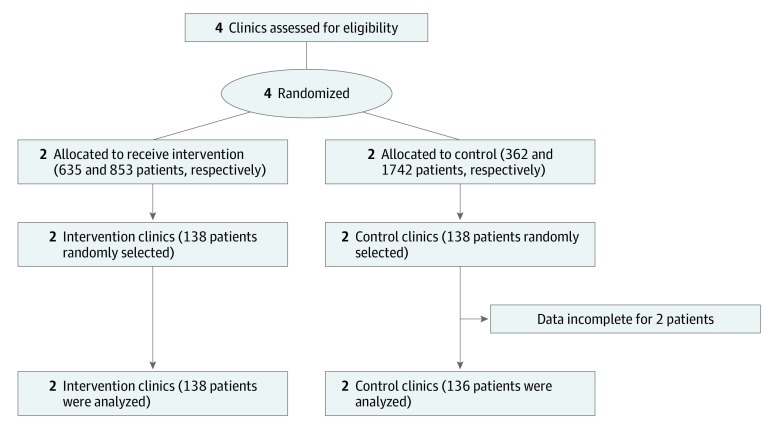
CONSORT Diagram for Cluster Randomized Trial Showing Randomization Allocation, Follow-up, and Analysis

Because cluster trials randomize interventions to groups of patients (eg, medical practices) rather than to individuals, 2 units of measurement, cluster and patient, are used. Each is reported. This study followed the Consolidated Standards of Reporting Trials (CONSORT) extension for cluster trials^24^ reporting guidance (Figure 1). This study was approved by the Indiana University Institutional Review Board, and parental consent was waived because of minimal risk and because consenting all children seen in the clinics without affecting study outcomes was impracticable. The trial protocol is available in Supplement 1.

### Intervention

The AAP policy statement regarding the identification and evaluation of children with ASD includes an algorithm describing when children should be screened and referred for a full evaluation.^25^ This algorithm was built into CHICA as an ASD module. CHICA is a rule-based system that has operated in primary care pediatric clinics at the Eskenazi Health System in Indianapolis, Indiana, since 2004.

CHICA has been described in detail elsewhere^26,27^; in brief, the system communicates with the underlying electronic health record (EHR) so that when a patient registers for care, CHICA analyzes the child’s EHR (demographic characteristics, morphometric characteristics, diagnoses, and medications) and selects the highest-priority 20 yes or no questions covering a wide range of primary care issues to ask the family. These are displayed on a sheet of scannable paper or an electronic tablet^28^ that is given to the family to complete in the waiting room. The questions are produced in English and Spanish. Our data show that approximately 90% of these forms are completed. CHICA analyzes the responses to these questions and selects the 6 most important alerts or reminders for the clinician. These are assembled into a visit agenda that can be printed on a scannable worksheet or displayed from within the clinician’s EHR. The clinician can respond to the alerts and reminders in the agenda by checking associated boxes in the EHR. These responses also store data that can be used for future decision support. Last, CHICA has a library of patient and physician handouts it can print as needed based on issues it has identified. CHICA covers a wide range of topics in primary pediatric care, from lead screening^20^ and asthma^19^ to adolescent depression^29^ and type 2 diabetes.^23^

The AAP ASD guidance was encoded in the CHICA ASD module by creating rules that directed both surveillance and screening.^30,31^ To conduct surveillance (as defined in the AAP guideline^25^), CHICA’s prescreening form asked parents whether they were concerned about the child’s development or whether the child had a sibling with ASD. If both of these were true or if 1 was true and the clinician had expressed developmental concerns in CHICA at an earlier visit, then CHICA produced an alert to the clinician that the child should be referred immediately for an ASD evaluation.

Otherwise, CHICA produced an M-CHAT-F ASD screening form.^12^ (In subsequent versions, CHICA was programmed to produce the M-CHAT-R/F screening form.) This form was originally printed on a barcoded sheet of paper that was completed in the examination room, scanned, and automatically scored by CHICA. In the newer version of CHICA, in which families completed the questionnaire on an electronic tablet, the M-CHAT-F (or M-CHAT-R/F) was displayed on the tablet and completed in the waiting room.

Through the EHR, CHICA alerted the clinician if the M-CHAT-F had a positive result. Moreover, CHICA printed the standard follow-up interview questions that were relevant to the items failed on the M-CHAT-F for the clinician to ask. The alert also asked the clinician to indicate in the EHR whether his or her assessment was concern for ASD and whether a referral to a diagnostic clinic was made. Because CHICA does not capture whether follow-up questions were used, we refer only to the M-CHAT.

At subsequent visits, CHICA prompted clinicians to indicate whether a diagnostic evaluation had been completed and what its result was. If a clinician indicated that the child was given a diagnosis of ASD, then CHICA implemented a series of monitoring questions for parents regarding financial concerns, behavioral concerns, need for respite care, and complementary and alternative therapies. If the parent indicated needs in these areas, then the clinician received an alert, and CHICA generated a handout to help the clinician and family navigate the issue.

Physicians and staff in the intervention group were told that CHICA would produce an M-CHAT screening form and that the form would be scored and the results provided to the physician through CHICA, including the relevant follow-up questions. No other training on ASD screening was provided except through the prompts in CHICA. No training was provided to the control group.

### Setting

This study took place in 4 primary care pediatric clinics in the Eskenazi Health System in Indianapolis, Indiana. The experimental intervention in 2 of these clinics included the enhanced version of the CHICA with the CHICA ASD module. The 2 control clinics also had CHICA, but it did not include the ASD module. Clinicians in control clinics identified and cared for children with ASD using their standard methods. Participants were automatically enrolled in the study based on which clinics they attended. Intervention clinic staff and physicians were instructed in the use of the CHICA ASD module during a 1-hour meeting. All 4 clinics had user support, consisting of a help desk and quarterly meetings with clinic teams to discuss general CHICA issues.

### Data Sources and Collection

To measure the effect of CHICA on ASD screening and surveillance, we assessed the percentage of children at the 18-month or 24-month visits who were screened using an ASD-specific screening tool between November 16, 2010, and November 21, 2012. Data were collected from 2 sources: a review of CHICA data and medical record abstraction.

#### Review of CHICA Data

As parents and physicians entered data directly into CHICA, information about the screening and diagnosis of ASD was collected automatically in the intervention clinics throughout the study period.

#### Medical Record Abstraction

Because physicians may have conducted screening without recording it in CHICA and because ASD screening data were not collected by CHICA in the control group, we used manual medical record abstraction to assess each clinic’s surveillance and screening rates related to ASD, independent of what was recorded in CHICA. Trained research assistants reviewed both the electronic medical record and paper records for a variety of information, including ASD screening and surveillance according to the AAP guideline.^25^ A random sample of medical records of eligible patients was abstracted at baseline and at 6, 12, 18 and 24 months after starting the intervention in both the intervention and control clinics. Research assistants were not blinded to group allocation. To assess the reliability of medical record abstraction, a 20% sample of the medical records was abstracted twice. Agreement on the primary outcome of ASD screening rates was substantial, with a κ of 0.79.^32^

### Participants

Children 3 years or younger who were seen in 1 of the 4 study clinics within 1 month of their 18-month or 24-month birthday were eligible for inclusion. Children born before 35 weeks’ gestational age and/or with a diagnosis of Down syndrome (trisomy 21) were excluded. The EHR was used to select eligible patients who had a visit to 1 of the clinics before the intervention started and to select samples at 6-, 12-, 18-, and 24-month intervals (±2 weeks) after the initiation of the intervention. Those who had a visit to the clinic when either 17 to 23 months of age or 24 to 36 months of age were eligible. A patient could have his or her medical record reviewed at only 1 time point. A random sample of eligible patients was identified.

### Outcome Measures

The primary outcome of interest was the percentage of children at the 18- or 24-month visits who were screened using an ASD-specific screening tool (ie, the M-CHAT). Data were also gathered to examine the following secondary outcome measures: (1) number of screened children who had a positive ASD screening result at the 18- or 24-month visit, (2) number of children with a positive screening who were referred for evaluation after the positive ASD screening result, (3) number of children who received a diagnosis of ASD after completion of a comprehensive ASD evaluation, and (4) percentage of children referred for audiologic evaluation after a positive ASD screening result.

### Power Calculations

We based our target sample size on the assumption that there would be a 10% screening rate at baseline and an increase to 40% in the intervention clinics. This gave us 90% power to detect at least this difference, setting α at .05, with a sample size of 49 per group at baseline and at follow-up. Because the unit of randomization was the clinic and the patients are nested within the clinic, we accounted for intracluster correlation by assuming screening rates varied from 6% to 12% by clinic. This translated to an intraclass correlation coefficient of 0.0041. To be conservative, we used an intraclass correlation of 0.0082, resulting in a sample size of 62 per group per time point.

### Statistical Analysis

Statistical analysis was performed between February 6, 2017, and June 1, 2018. The primary outcome, percentage of eligible children screened for ASD, was evaluated first with a run chart showing the percentage of the sample screened at each time point (baseline and 6, 12, 18, and 24 months after initiation of the CHICA ASD module), with separate lines for each group. Screening rates at each time point were compared by means of the Fisher exact test. All *P* values were from 2-sided tests and results were deemed statistically significant at *P* < .05. To control for intraclass (within-clinic) correlations, we compared the proportion of children screened between the intervention and control groups by modeling the postintervention outcome (screened: yes or no) using a logistic model with a term for group and an exchangeable correlation structure that adjusted for the correlation of children from the same clinic. Because of imbalanced race/ethnicity in the intervention and control groups, this analysis also controlled for race/ethnicity. The postintervention data at different time points were combined for modeling. Secondary outcomes were descriptive and are presented in the text as proportions with 95% CIs.

Analyses were performed with SAS/STAT software, version 9.4 (SAS Institute Inc). Graphs were produced using R Core Team, version 2015 (R Project for Statistical Computing).

## Results

At the request of clinic leadership, the 18-month M-CHAT was stopped within 6 months of starting the intervention. Physicians believed it was overwhelming for families to complete the M-CHAT at the 18-month visit in addition to the Ages and Stages Questionnaire developmental screening, which the clinics were also using according to AAP recommendations. CHICA continued to screen for ASD with the 24-month M-CHAT.

### M-CHAT Test Positivity

By the end of the study, 40 820 children 21 months or younger had visits using the CHICA system in the 4 study clinics, 34.0% (n = 13 871) in intervention clinics and 66.0% (n = 26 946) in control clinics. During the intervention, M-CHAT screening tests were printed for 1653 children, aged between 20 and 36 months. Of the 1653 M-CHAT tests printed, 980 (59.3%) were completed and scanned back into the system for scoring by CHICA. We suspect that some physicians also scored M-CHAT tests manually. Scored M-CHAT tests showed that 265 children had results possibly indicative of ASD, for a 27.0% positive screening rate.

### Effects of CHICA on ASD Screening

Abstractions were completed on 274 medical records: 129 at baseline, 38 at the 6-month time point, 36 at the 12-month time point, 35 at the 18-month time point, and 36 at the 24-month time point after initiating the intervention. By design, these were evenly divided among intervention clinics (n = 138) and control clinics (n = 136).

The sex, race/ethnicity, and insurance status of the children included in the medical record abstraction are shown in the Table. All children were between 23 and 30 months of age by design. There were more boys (n = 162) than girls (n = 101). Most children (242 of 263 [92.0%]) were covered by Medicaid. Most children (244 of 263 [92.8%]) were nonwhite. Overall, 138 (52.5%) were African American; 96 (36.5%) were Hispanic. There was a larger Hispanic population in the control group than in the intervention group (91 of 136 [66.9%] vs 5 of 127 [3.9%]; *P* < .001) and a larger African American population in the intervention group than in the control group (106 of 127 [83.5%] vs 32 of 136 [23.5%]) (*P* < .001).

**Table.** Sex, Race/Ethnicity, and Insurance Coverage of Children^a^

Characteristic	Children, No./Total No. (%)		
	Overall (N = 274)	Intervention (n = 138)	Control (n = 136)
Sex			
Female	101/263 (38.4)	43/127 (33.9)	58/136 (42.6)
Male	162/263 (61.6)	84/127 (66.1)	78/136 (57.4)
Race/ethnicity			
Asian	4/263 (1.5)	2/127 (1.6)	2/136 (1.5)
Native American	2/263 (0.8)	2/127 (1.6)	0
Black	138/263 (52.5)	106/127 (83.5)	32/136 (23.5)
Hispanic	96/263 (36.5)	5/127 (3.9)	91/136 (66.9)
Unknown	4/263 (1.5)	1/127 (0.8)	3/136 (2.2)
White	19/263 (7.2)	11/127 (8.7)	8/136 (5.9)
Insurance			
Commercial	10/263 (3.8)	4/127 (3.1)	6/136 (4.4)
Medicaid	242/263 (92.0)	119/127 (93.7)	123/136 (90.4)
Self-pay	9/263 (3.4)	3/127 (2.4)	6/136 (4.4)
Special payer	1/263 (0.4)	1/127 (0.8)	0
Unknown	1/263 (0.4)	0	1/136 (0.7)

^a^ Missing values are excluded.

**Figure 2.**
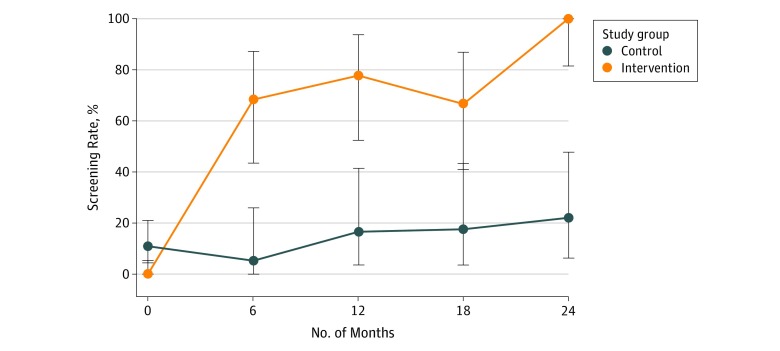
Run Chart Showing the Rates of Autism Spectrum Disorder Screening in Eligible Children During the Study Period The screening rate at each time point for each group was estimated using the binomial distribution, and the 95% CIs (error bars) were from Clopper-Pearson (exact)–type intervals.

The primary outcome of the study was the rate at which eligible patients were screened for ASD using a standardized screening instrument such as the M-CHAT. This rate increased over time in the intervention group but not the control group. At baseline, none of the children in the intervention group were screened. In the control group, 7 of 64 children (10.9%) were screened. During the intervention period, 57 of 73 children (78.1%) in the intervention group were screened. The intervention clinics’ screening rates increased from 0% (95% CI, 0%-5.5%) at baseline to 68.4% (13 of 19) (95% CI, 43.4%-87.4%) at 6 months to 100% (18 of 18) (95% CI, 81.5%-100%) at 24 months. The control group screening rate during the study period was only 15.3% (11 of 72 children) at 6 to 24 months after the intervention, peaking at 4 of 18 children (22.2%) at 24 months. Differences between groups became statistically significant during the intervention period (Figure 2). Comparisons controlling for intraclass correlation showed that intervention clinics were much more likely to screen children for ASD (odds ratio, 108.23 [95% CI, 22.65-517.2]).

**Figure 3.**
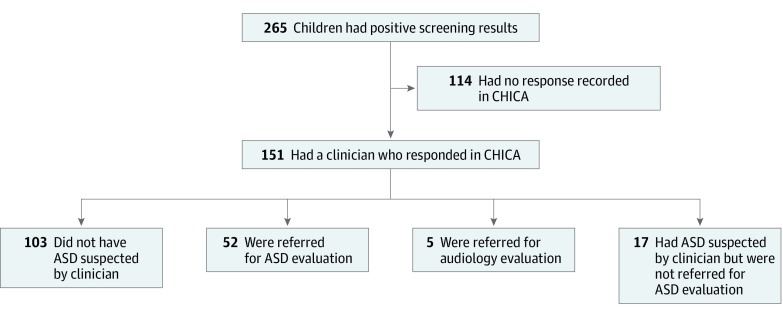
Physician Responses to Alerts Indicating Child Had a Concerning Modified Checklist for Autism in Toddlers Result Total percentages exceed 100% because physicians could check more than 1 response per child. ASD indicates autism spectrum disorder; and CHICA, Child Health Improvement Through Computer Automation system.

Despite the increased ASD screening, clinics and physicians were not as effective in following up when patients had positive screening results. Among the 265 patients with a positive M-CHAT result, physicians indicated any response to the positive M-CHAT result for only 151 children (57.0%; 95% CI, 51.0%-62.9%). In 103 of the 151 with responses (68.2%; 95% CI, 60.8%-75.6 %), pediatricians indicated the child did not have ASD, 52 of 151 children (34.4%; 95% CI, 26.8%-42.0%) were referred for ASD evaluation, 17 of 151 children (11.3%; 95% CI, 6.2%-16.3%) were suspected of having ASD but not referred, and 5 of 151 children (3.3%; 95% CI, 0.4%-6.2%) were referred for audiologic evaluation (Figure 3).

Medical record abstraction showed that, although children in intervention clinics were more likely to be screened for ASD, physicians in those clinics were less likely to document screening results when they were positive (odds ratio, 0.18 [95% CI, 0.02-1.89]). Nonetheless, full referral and evaluation for ASD were more likely to occur in the intervention group (odds ratio, 19.88 [95% CI, 3.33-118.65]).

By the end of the study period, 52 children had documentation in CHICA of referral for an ASD evaluation. Two of 138 children (1.4%) in the intervention group had a new ASD diagnosis recorded during the intervention by medical record review. Among all children screened by CHICA, 15 of 980 (1.5%) received an ASD diagnosis. Because 15 children were identified as having ASD among the 151 children with positive screening results who were evaluated, the positive predictive value of the M-CHAT could be estimated at 10%.

## Discussion

Automated screening and clinical decision support had an effect on the rate of routine screening for ASD in general pediatric practice. Screening in the intervention clinics went from 0% to 68.4% within 6 months and then to 100% during the 24 months of the study. This outcome, while larger, is consistent with previous work on clinical decision support systems.^17,22,33^ It is not clear why the rate increased over time, but previous work with CHICA shows that physicians seem to become accustomed to decision support over time.^34^ Automated systems that screen for ASD have been described that also score the follow-up interview in real time and could improve physician response rates.^35^ However, this study is the first, to our knowledge, to rigorously test the effectiveness of such systems for improving rates of screening consistent with authoritative guidelines.

The control clinics had access to the M-CHAT-F tool but to no other systematic approach to increase screening. Therefore, the CHICA module was not compared with a competing improvement strategy. Daniels et al^36^ conducted an extensive review of approaches to improving early detection of ASD. Among the 40 studies reviewed, a small amount were conducted in primary care, included ASD screening, and assessed rates of screening. Only 1 study was a randomized clinical trial, and it achieved a screening rate of 81%. Most studies assessed only postintervention screening rates, which varied between 80% and 90%. One study, which assessed screening rates before and after “academic detailing,” achieved a 71% screening rate.

Our results show that automating surveillance for ASD and automating administration of a screening test can result in very high rates of screening. This method also has the advantage of ensuring that scoring is done correctly. The weak point in the process appears to be the clinical response to screening results, as evidenced by the apparent nonresponse of physicians to almost half of positive M-CHAT results.^30^ This finding is consistent with previous work on clinical decision support systems.^37,38^ In fact, the greater than 50% response rate achieved by CHICA is high compared with other types of physician alerts, which may be ignored more than 90% of the time.^39^ We believe that embedding the decision support within a visit agenda significantly improved this response rate. Therefore, even with this weak point, screening and subsequent evaluation for ASD were improved with CHICA’s ASD module.

Two new diagnoses of ASD (1.4%) were documented in the medical records reviewed. Physicians reported 15 ASD diagnoses (1.5%) among all children exposed to the CHICA module. These percentages are slightly lower than the estimated prevalence of ASD, but limited time and local diagnostic capacity may explain this finding. The study was not powered, nor was it of sufficient duration, to detect a difference in rates of diagnosis of ASD. We may anticipate that ASD detection will improve, but the 57.0% response rate from physicians, combined with the poor follow-through by parents,^40^ will attenuate the effect of universal screening.

### Limitations

This study has limitations that warrant consideration. The study did not show adherence to the AAP guidelines to screen children at 18 months and did not show that ASD screening and developmental screening can be conducted at the same visit per AAP recommendations. The study was confined to a small number of clinics. Its randomized design, however, was powered to detect an intervention effect much smaller than we found. The randomization failed in that the racial/ethnic makeup of the intervention and control clinics differed. However, these differences were controlled for in the analyses, and the inclusion of baseline and postintervention data shows that this difference is unlikely to explain the results.

The population of patients in the intervention clinics was 83.5% African American. Racial minority groups, especially African American individuals, are less likely to be screened for and receive a diagnosis of ASD.^41^ It is notable, therefore, that screening and follow-up rates were increased in this group. Unfortunately, the clinics thought it was not feasible to conduct both the M-CHAT and the Ages and Stages Questionnaire at the same visit. Resolving this issue may require changes in the clinic workflow or for families to complete these procedures at home (eg, through an online patient portal).

Another challenge to screening with the M-CHAT-F and M-CHAT-R/F is the high rate of positive screening results (27.0% in our study). This finding is comparable to positive screening result rates in similar populations described by Daniels et al.^36^ The follow-up questions in the M-CHAT-F are intended to reduce the false-positive rate. CHICA automatically produced follow-up questions, but we do not know how well they were used. The high false-positive rate means that most children with positive screening results will not have ASD, and it has been suggested, therefore, that referral is unjustified.^42^ In fact, since 15 children were identified as having ASD among the 151 children with positive screening results who were evaluated, the positive predictive value of the M-CHAT could be estimated at 10%. This finding, too, may explain why many physicians did not respond to positive screening results.

Therefore, screening for ASD is but the first step in improving outcomes for children. Too often, there are insufficient resources available to make proper diagnoses of ASD and even fewer resources available to treat children with a diagnosis of ASD. Improving those factors will be necessary to improve the outlook of the many children in the United States who have ASD.

## Conclusions

Automation, as with the CHICA system, which integrates into routine care and ensures that screening is administered to most eligible patients, can drastically improve the rates at which children are screened for ASD. This automated screening is necessary, but not sufficient, to improve the care of children. More work is needed to automate the further evaluation of children who screened positive for ASD.

## Notes

Supplement 1. **Trial Protocol**

Supplement 2. **Data Sharing Statement**
