# Supplementary material for: Effect of a Computer-Based Decision Support Intervention on Autism Spectrum Disorder Screening in Pediatric Primary Care Clinics: A Cluster Randomized Clinical Trial
Source: JAMA Netw Open. 2019 Dec 18;2(12):e1917676. doi: 10.1001/jamanetworkopen.2019.17676 (PMC6991212; doi:10.1001/jamanetworkopen.2019.17676)
Supplement: Supplement 1. — Trial Protocol [file jamanetwopen-2-e1917676-s001.pdf]

D. Research Design and Methods

This will be a randomized trial in which we will compare changes in screening, diagnosis, and management of ASD before and after implementation of the CHICA system in intervention control practices. Although the unit of randomization will be the primary care practice, the unit of analysis will be the individual patient.

Currently, CHICA is being utilized within a purely academic setting. However, by the start of this research project, CHICA will have expanded into the community practice setting through its incorporation into pediatric clinics in the Indianapolis area that participate in the Indiana University Medical Group – Primary Care (IUMG-PC) practice network. IUMG-PC includes 14 clinic sites that cater to pediatric patients. In 2007, these clinics hosted 95,011 patient visits for children between the ages of 0 and 13. 24% of these visits were for pediatric patients with Medicaid.

For this study, we will be focusing on four of the pediatric IUMG-PC clinics that have been previously set up with the CHICA system. These four clinics see the largest number of pediatric patients within the IUMG-PC practice network. For example in 2007, these four clinics saw 8,528 patients between the ages of 0 and 3 years and 3,065 patients between the ages of 4 and 5 years. Two of the four clinics will have the ASD modules turned on; two will not and will function as the control group. This will allow us to test the impact of the specific ASD modules above that of CHICA alone. The four clinics will be ranked by their size, where size is defined by the number of physicians and residents in the clinic. A randomization plan will be used such that the first and last ranked clinics will be assigned, at random, to one group (intervention or control) and the other two clinics will be assigned to the other group.

Although randomization at the physician/patient level is sample-size efficient, we chose a cluster randomization by clinic because contamination is a major concern. If we randomize at the physician level, physicians in the same clinic who are assigned to different treatment arms might communicate regarding the CHICA system in terms of its operation and consequences, which could contaminate the control arm. If we randomize at the patient level, the on and off usage of the CHICA might confuse the physician and lead some to think that CHICA is malfunctioning. Anecdotally, this has been our experience with the asthma and maternal depression studies we have conducted. We have found previously that randomization at the patient level can lead to irritation and inconvenience for both physicians and clinic staff. The limitation of randomization at the clinic level is inflated sample size and potential bias caused by unknown characteristics that are differentially distributed in the four clinics and related to the outcome. Characteristics of this nature could include awareness of ASDs by physicians, socio-economic status of patients and so on. To our knowledge however, there is no reported significance and scale of the effect of these factors. After weighing the limitation and advantage of each option, we decide to allocate two clinics to each of the study arms. We will adjust for intra-cluster correlation and potential confounders in the analysis.

D.1 Expand and modify an existing computer-based decision support system (CHICA) to assist pediatricians with identification and management of ASDs through the addition of the ASD screening and surveillance module and the ASD care and management module. (Aim 1):

The CHICA system has been a successful prototype in the academic setting. Several enhancements are needed, however, in order to incorporate it into everyday use in pediatric office practices. Some of these are technical in implementation, some are functional. Many of our proposed changes are based on our experience, as reported in the literature (see section B.3); others arose during system development, clinic use, and evaluation, as we faced roadblocks in implementation, or when we recognized new potential applications. Other ideas came from users who were either unhappy with some component of the system or identified new areas where CHICA could be improved. Maximizing CHICA's utility will increase its impact on child health and enable the Autism diagnosis and treatment parameters to be added to the system. Enhancements will be completed in the first year of the project.

D.1.1 Program CHICA to automatically evaluate M-CHAT forms

One of CHICA's greatest strengths is its ability to automate the collection and analysis of large amounts of data for physicians. In this case, we will use CHICA to score the forms related to ASD screening, diagnosis and management. For example, CHICA will generate the Modified Checklist for Autism in Toddlers (M-CHAT) form for parents to fill out at their child's 18 to 24 month visit. This form will then be scanned into CHICA, where it

will be automatically scored by the computer system. This information will then be given back to physicians on the provider worksheet along with recommendations for next steps in those children who screen positive for ASD. All data can be grouped by patient and analyzed longitudinally.

<u>D.1.2 Program CHICA to include an algorithm to assist pediatricians in the early identification of ASDs</u>
ARDEN rules will be generated to use all of the collected data to recommend care for each child in the intervention arm of the study. Specifically, we will take the American Academy of Pediatrics (AAP) algorithm that has been created for Autism surveillance and screening[5] and generate CHICA rules, using methods already developed in previous work (C.2.1). The overall flow of the CHICA ASD surveillance and screening module that will be used in the intervention arm of the study is pictured in Figure 8.

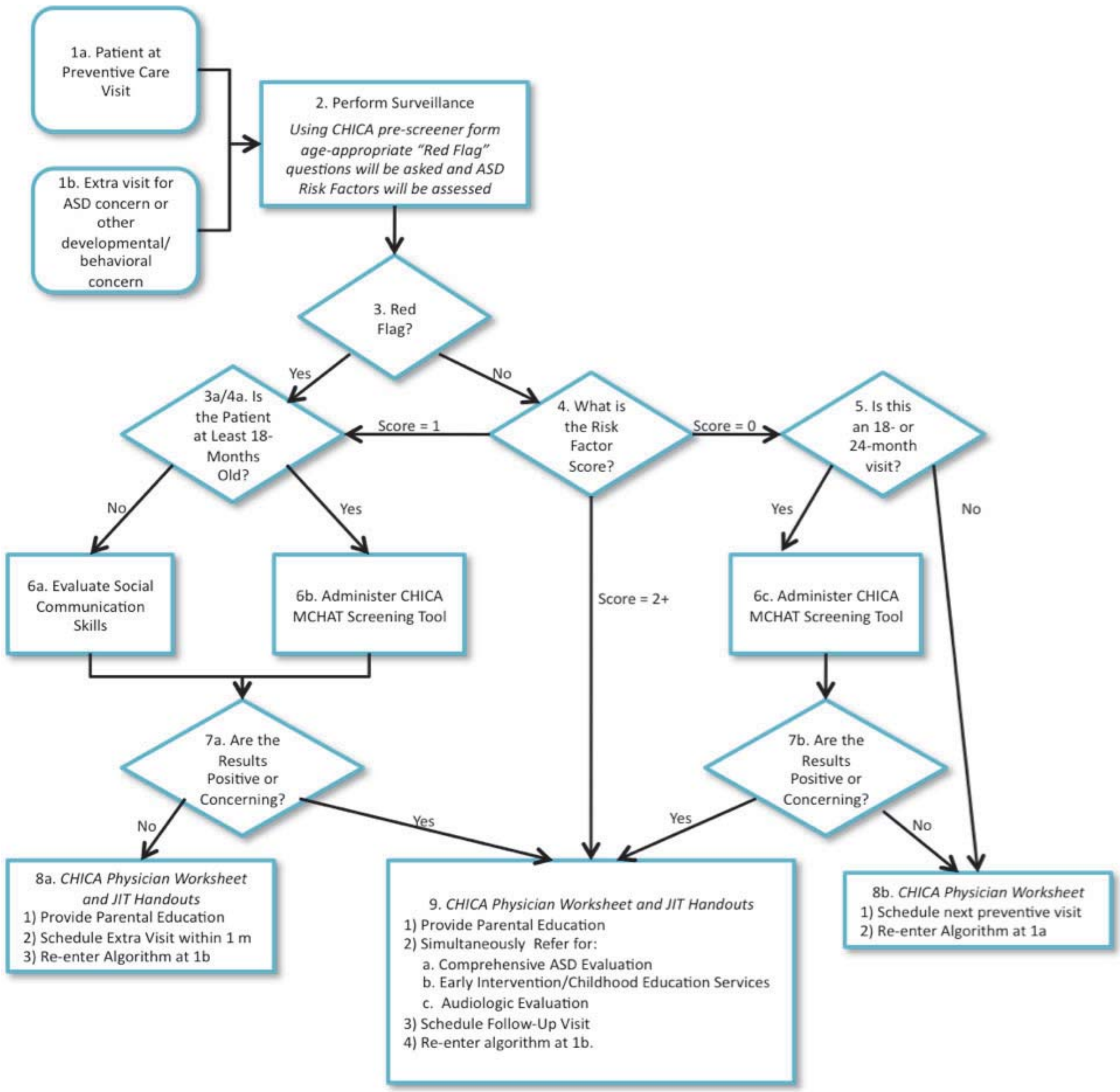

Figure 8. Flow of the CHICA ASD Surveillance and Screening Module

<u>D.1.3 Program CHICA to include management and treatment recommendations to guide pediatricians in the care of children with ASDs.</u>
Rules will also be written to assist pediatricians in the management of children suspected of having an ASD. This includes rules for suggested referrals and scheduling of follow-up appointments. We will also create rules to oversee practice in general, remind physicians when patients have missed visits, and when forms have not been turned in. CHICA will also generate just-in-time forms when appropriate in order to continually evaluate behavioral progress in each participant and to provide educational materials aimed at parents. This will

provide data for both the evaluation of current care and for necessary changes in the care regimen that may improve care. Specific components of the ASD care and management module is further described in section D.3.

D.1.4 Validation of the CHICA ASD modules

All CHICA rules and rule sets undergo thorough testing. We will first review the logic behind them in groups, to make sure that they appear valid. We will then proceed to simulations, running them with test patients to make sure that they are firing accurately. The final ASD rule set will be reviewed first by our investigators (pediatricians and a psychologist). Then the rule set will be reviewed by the Pediatrics Practice Advisory Committee (PPAC), a group of pediatricians in Indiana University Medical Group who develop and validate all practice guidelines for the participating community practices in the group. After rule deployment, we meet monthly with the CHICA User's Group, and review all CHICA process data in weekly team meetings to make sure that randomization and rule firing appears valid. Should any issues arise, we can implement fixes in a timely manner. This type of review goes on continuously and would be part of the study process as well.

D.1.5 Implementation of the CHICA system and ASD modules

We have extensive experience implementing the CHICA system and are aware of many potential barriers/pitfalls that can be encountered during deployment. Therefore we have developed a process to overcome these potential issues and to maximize the benefit of the technology. When we are ready to implement CHICA in the new sites, a technical team of lead programmers and pediatric informaticians will visit the clinic and conduct the following steps with members of the clinic, including physicians, nurses, clerical staff, and clinic leadership:

1. The CHICA system will be presented to the clinic.
2. With assistance from the CHICA team, the nursing and clerical staff will create workflow diagrams of the clinic, tracking flow of patients and medical records.
3. The CHICA team and the clinic staff will develop a plan for inserting CHICA into the workflow, including who will print forms, hand them to the patients, fill them out, and scan them into the system.
4. After CHICA is installed, the CHICA team will have a dry run – using the system without the clinic staff for one week to assure reliable technical function.
5. Clinic staff will receive a brief (<1 hour) training on how to fill out the forms and operate the scanner, as well as how to page CHICA staff if needed.
6. Clinicians will receive a 15-minute presentation about CHICA, how it works, and how to fill out forms.

We have (and will continue to have) one programmer who is always on call to perform technical support. In addition, a less technical member of the CHICA team will rotate full time through the clinics to assess CHICA's use and note any issues or problems for the full team to address. Further, we have established a CHICA user's group (CHUG), where the CHICA team leader (Downs) and one of the programmers meets in the clinics over breakfast or lunch on a regular schedule to allow clinic personnel to address members of the CHICA team. We have found that this installation and training package is sufficient to get CHICA up and running.

D.2 Evaluate the effect of the CHICA system ASD screening and surveillance module on the early identification of ASD in four pediatric practices (Aim 2).

Through questions about ASD symptoms on the pre-screener form, we expect that patients with ASD will be more readily identified by the clinics with the CHICA ASD surveillance and screening module. This ASD module will also allow CHICA to prominently display messages on the physician worksheet. Our prior work has shown that these efforts are successful in helping the caregiver to identify patients who require attention.

D.2.1 Intervention Group:

The experimental intervention will include the enhanced version of the CHICA hardware and software, the CHICA ASD surveillance and screening module, and user support. Because the intervention is at the clinic level, all patients will be automatically cared for by the CHICA system and the CHICA ASD surveillance and screening module in the two intervention practices as part of routine medical care. The 2007 AAP surveillance and screening algorithm for ASDs addresses the care of children through the first 5 years of life. Therefore this is the target patient population for our intervention. The actual intervention will have a number of components that we expect will improve the surveillance and screening practices of pediatricians for ASDs:

1) *Case finding* – On the pre-screener form, parents will be asked simple questions in order to identify "red flags" that are indications for immediate evaluation (See Figure 8 Box 2). These include:
    - No babbling or pointing or other gesture by 12 months
    - No single words by 16 months
    - No 2-word spontaneous (not echolalic) phrases by 24 months
    - Loss of language or social skills at any age

    Questions will also be asked to assess risk factors for ASD. These include:
    - A sibling with ASD
    - Parental concern
    - Other caregiver concern
    - Pediatrician concern

    Each of these four risk factors is worth one point. If a child does not have a sibling with ASD and there are no concerns from the parent, caregivers, or pediatrician then a risk factor score of zero will be given. If the child has either a sibling with ASD or concern is expressed by a parent, caregiver, or pediatrician, then the child is given a risk factor score of 1. If the child has 2 or more risk factors then they receive a score of 2+ (See Figure 8). Based upon these concerns, screening might occur in the examination room. Screening would be done through the use of a printed M-CHAT form. These forms would be automatically scored by CHICA. Moreover, universal screening will occur at the 18- or 24-month visits through the use of an M-CHAT form printed at patient check-in. At these visits, all parents will be given a preprinted M-CHAT to fill out for their child in the waiting room (See Figure 8).

    The M-CHAT[24] is a simple screening tool that can be given to children during pediatric visits. It does not rely on the physician's observation of the child, but on parents' report of current skills and behaviors. The tool contains 23 yes-no items. Initial failure on the screener is defined as any three items failed, or any two critical items failed. The critical items were identified by discriminant function analysis of children with and without a disorder on the autism spectrum and included items concerning joint attention (proto-declarative pointing, bringing to show, following a point), interest in other children, responding to name, and imitation.[57] The sensitivity is reported to be 87% and the specificity 99% in the referred sample (i.e., children already identified as having developmental concern).[58, 59] The M-CHAT was chosen due to its simplicity and the fact that it does not require physician administration.

    The ASD surveillance and screening module will also allow CHICA to prominently display messages on the physician worksheet. Data that can be displayed on the physician worksheet includes whether parents have any Autism-related concerns about their child as identified on the pre-screener form and/or M-CHAT scores from universal screening that will occur at either the 18 or 24-month visit. Also if a child demonstrates a failure on the M-CHAT screener, follow-up questions would be displayed on the physician worksheet or via just-in-time documents to clarify and elicit specific examples of the child's typical behavior relevant to each failed M–CHAT item.

2) *Diagnosis* – Because a positive screening result does not determine a diagnosis of ASD, any child who receives such a result must be referred for comprehensive ASD evaluation. Based on the child's ability to refer for testing, this evaluation should include:
    - 3-generational family pedigree with health, developmental, and behavioral histories
    - Thorough physical exam with Wood's lamp skin examination
    - Developmental and/or psychometric evaluation
    - Evaluation with standardized tools for presence of categorical DSM-IV-TR diagnoses
    - Assessment of parental knowledge of ASD and appropriate resources and skills
    - Laboratory investigation for etiologies or known coexisting conditions

    Additionally, these children should also be referred to early intervention/early childhood education services (depending on the child's age) and receive an audiologic evaluation. CHICA will make explicit recommendations to the physician on the physician worksheet to provide these referrals if appropriate and the system will also provide the proper forms so that such referrals and evaluation can be arranged (See Figure 8 Box 8).

3) *Reassessment* – CHICA will automatically track those who have been previously screened. According to the algorithm (See Figure 8), those who previously have been assessed should be automatically assessed at subsequent visits. CHICA can insure that participants are not lost to follow-up.

The overall flow of the CHICA ASD surveillance and screening module is pictured in Figure 8 and closely resembles the surveillance and screening algorithm for ASD recommended by the AAP.[5]

D.2.2 Control Group

The two control clinics will also have the CHICA system in place and, therefore, all patients in the control clinics will be automatically cared for by the CHICA system. However, the CHICA system within the control clinics will *not* include the ASD surveillance and screening module. Instead, clinicians in these clinics will screen and diagnose children for ASDs using their standard methods of care. These control clinics will however receive paper copies of the 2007 AAP Surveillance and Screening Algorithm for ASDs.[5]

D.2.3 Patient Recruitment and Consent

Four pediatric clinics in the IUMG-PC practice network will already be equipped with the CHICA system prior to the beginning of this research project. For this particular study, we will add the ASD surveillance and screening module to the CHICA system in two of these clinics (i.e., intervention clinics). The other two clinics will not be provided with the ASD surveillance and screening module (i.e., control clinics).

We will ask for a waiver of consent for this portion of the study as 1) there is little risk involved in supplying physicians with guidelines of care, 2) study procedures are within standards of usual care, 3) informing families that they may be part of an ASD study would bias their response to study questions and invalidate any and all results of the project, and 4) obtaining informed consent from the many thousands of patients who are seen at IUMG-PC clinics would be difficult and would itself create, because of the paperwork that would be required, a much higher risk of loss of patient confidentiality.

D.2.4 Data Collection

In order to evaluate the effect of the CHICA system on the screening and diagnosis of ASDs, data will be collected from two sources:

1. *Medical record abstraction.* We will use chart abstractions to assess each clinic's surveillance and screening practices related to ASDs. Research assistants (RAs) will be trained to review both the electronic medical record and paper charts for a variety of information including how often ASDs are screened for and how often surveillance is performed according to the 2007 AAP algorithm. To assess the reliability of chart abstraction, a random sample of 20% of the charts will be abstracted twice.

2. *Review of CHICA data*. Based upon information provided by physicians on the physician worksheet, CHICA will automatically collect data on the screening and diagnosis of ASD throughout the study period. Moreover, we can add questions to the pre-screener form to ask parents if their children have ever been diagnosed with an ASD.

Additionally, information about practice characteristics will be collected at baseline. The medical record abstraction and review of CHICA data will be conducted at baseline and at six-month intervals until study completion. These methods will allow a thorough description of the intervention's role in the process of ASD surveillance and screening.

D.2.5 Outcome Measures

The primary outcome of interest for aim 2 is the percent of children at the 18 or 24-month visits who are screened using an ASD specific screening tool (i.e., M-CHAT).

Data will also be gathered in order to look at the following secondary outcome measures:

- Percent of children under the age of 5 undergoing surveillance for ASDs (See Figure 8 Box 2)
- Percent of children where ASD surveillance led to screening with an ASD specific tool (i.e., M-CHAT)
- Percent of children who had a positive ASD screening result at the 18 or 24-month visit

- Percent of children who were referred for comprehensive ASD evaluation after positive ASD screening result
- Percent of children diagnosed with an ASD after completion of comprehensive ASD evaluation
- Percent of children referred appropriately to early intervention/early childhood education services after positive ASD screening result
- Percent of children referred for audiologic evaluation after positive ASD screening result.
- Mean and median age of children diagnosed with an ASD
- Percent of children re-screened appropriately based on AAP algorithm

Surveys will be administered at baseline and yearly to the entire staff of each practice, including physicians, clinical staff, administrative staff, and office managers in order to document how each practice is organized and provide detailed information about knowledge and attitudes about ASD screening, diagnosis, and management. Questions regarding knowledge and attitudes about ASD will be taken from the Autism Survey which is currently utilized by researchers with the HANDS in Autism Team at the Christian Sarkine Autism Treatment Center who are involved in this study. This survey was adapted from a previous survey instrument utilized by Stone.[60] Staff will also be asked about their satisfaction with the CHICA system and the ASD modules.

D.3 Collect early data on the effect of the CHICA system on services provided to children diagnosed with ASD and how these affect patient outcomes. (Aim 3)

At this time, the services that should be provided for children with ASD are not stipulated; moreover, the effect of individual services on patient outcomes is not well understood. However, there is a general sense that services should be given as early as possible, and that more services are better. We will therefore take advantage of the CHICA system to gather data on how the system affects the number of activities/involvements that children with ASD receive and how it affects patient outcomes. The data collected may provide information on how services affect outcomes and on how CHICA affects both. These data will be instrumental in further studies of both the CHICA system and autism care, in general.

D.3.1 Intervention Group

The intervention will include the enhanced version of the CHICA hardware and software, the CHICA ASD care and management module, and user support. Because the intervention is at the clinic level, all patients qualifying for the study will be automatically cared for by the CHICA ASD care and management module as part of routine care in the intervention practices. The CHICA ASD care and management module will provide the pediatrician with a number of recommendations and reminders that we expect will improve the management of patients diagnosed with an ASD. CHICA can prompt physicians to check that children with ASD are receiving the necessary services that will maximize their potential and quality of life. These can include:

- *Educational Interventions*. Specific and age-appropriate interventions could include comprehensive programs for young children inclusive of strategies and methods appropriate to ASD and evolving from applied behavior analysis, structured teaching, developmental models, speech and language therapy (e.g., conversational skills, social-relating skills), occupational therapy (e.g., fine motor and sensory integration methods)
- *Medical Management*. Like all children, those with ASD need basic health promotion and disease prevention care, such as immunizations. However, further care should be paid to the management of seizures, gastrointestinal problems, sleep disturbances, and challenging behaviors
- *Family Support*. Families play a key role in the care and management of children with ASD, and support should be given for education, emotional support, and advocacy for their children's needs

Based on the best available evidence CHICA can guide physicians through what activities and involvements should be considered for inclusion in a treatment and management program for children diagnosed with ASD. Examples of these are provided in Figure 9

- **Speech Therapy** (focused on pragmatic and social skills)
- **Occupational Therapy** (focused on fine motor and sensory issues)
- **School District Autism Consultant** (if appropriate)
- **Developmental Physician and/or Psychiatrist**
- **First Steps and/or Special Education Services**
- **Behavioral Therapist** (for parent education and/or intervention)
- **Medicaid** (3 relevant waivers in the State of Indiana)
- **Neurology** (Evaluation and treatment of concomitant seizure/neurological disorders)
- **Genetics** (Rule out of comorbid and/or contributing diagnoses)
- **Audiology** (Rule out hearing impairment)
- **Gastroenterology** (Management of intestinal issues)
- **Individualized Education Plan**
- **Behavior Intervention Plan or Individualized Family Support Plan**

Figure 9. Activities/Involvements Recommended for Children with ASD

D.3.2 Control Group

Control clinics will also have the CHICA system in place, and therefore all patients in the control clinics will be automatically cared for by the CHICA system. However, the CHICA system within these control clinics will NOT include the ASD care and management module. Care for children diagnosed with ASD will use standard methods.

D.3.3 Eligible Patients

Eligible patients within each practice include children who are seen in one of the four clinics participating in this study. For evaluating the management of ASD only children ages 18 months through 5 years with newly diagnosed ASD will be eligible for the study. Additionally, at least one of the child's parents must speak English and be available to be contacted by telephone.

D.3.4 Patient Recruitment and Consent

The same four clinics utilized in aim 2 of the study will be utilized for aim 3 of the study. The same two clinics that served as the intervention group in aim 2 will once again serve as the intervention group for this portion of the study. These two clinics will have the ASD care and management module as part of the CHICA system. The two control clinics will have the CHICA system but will NOT have the ASD care and management module.

For this portion of the study, consent will be obtained from those patients who are newly diagnosed with ASD so that we may collect additional evaluation and assessment data beyond that which is normally documented during standard care practices. Children and their parents will be recruited and consented through the Pediatric Research Network (PResNet). PResNet is a pediatric practice based research network located at the Indiana University School of Medicine (See full description in Resources section of the proposal). PResNet was established in 2006 as an offshoot of ResNet, the very successful practice-based research network of the Indiana University Medical Group. Since 2000, ResNet has been involved in more than 40 studies supported by over $15 million in extramural grants. In these studies, ResNet has recruited more than 7,000 patients, approximately 68% of those patients contacted (a remarkable figure, considering that two-thirds of those approached were African-American or Hispanic). In 2006, PResNet was established to facilitate pediatric specific primary care research efforts. While it does not have as long of a track record of recruiting patients as ResNet, it follows the same processes and procedures and therefore has encountered the same level of success at patient recruitment. PResNet has been involved with 8 studies in the IUMG clinics and has recruited more than 2200 patients, approximately 84% of those patients contacted. With the inclusion of the private practices, PResNet has recruited over 5000 patients, approximately 85% of those patients contacted.

Each week, the PResNet RA will be given a list of potentially eligible children who were diagnosed with ASD in one of the four study clinics during this study. Children will be identified by looking for ASD diagnostic billing codes or diagnostic codes using the RMRS. The RA will confirm the diagnosis with the physician and then contact the child's parent via telephone in order to assess eligibility, describe the study, and invite their participation. PResNet has a business associate's agreement (BAA) with the Indiana University Medical Group and is, in fact, the research arm of that organization. Therefore, the PResNet RA will introduce him/herself as calling from the child's doctor's office on behalf of the doctor. If the parent expresses interest, the PResNet RA will arrange to meet with them preferably at their next clinic visit. After the child checks into the clinic for their appointment, the RA will talk with the child and parent in a private space, usually an exam room. At that time, the RA will assess eligibility, administer the HIPAA authorization, complete the informed consent form and collect additional contact information.

D.3.4 Data Collection

To evaluate the effect of the CHICA system on the management of ASD patient we will collect data related to various process and outcome measures with our primary outcome of interest for aim 3 being the number of activities/involvements that a child diagnosed with an ASD is receiving.

Process Measures. Listed in Figure 9 are examples of activities and involvements that should be considered for inclusion in a treatment and management program for children diagnosed with ASD. We will create a checklist of these various activities and involvements that will be used to evaluate the individual components of a child's ASD management program (Bauer and Swiezy). We will collect these data at six months and 12 months after diagnosis from reviews of the medical record and short 15 to 30 minute interviews with parents. We anticipate that those patients cared for in the two clinics with access to the CHICA ASD care and management module will demonstrate a higher number of these recommended activities/involvements.

Outcome Measures. We will also collect data on a variety of patient outcomes related to ASD care and management. These assessments (Table 1) will be conducted with study participants at baseline, 6 months, and 12 months after diagnosis. PResNet RAs will be trained by experts in ASD (Bauer and Swiezy) on how to administer these measures to parents of participants in both arms of the study. These assessments will measure changes in the child's behavior, problem situations, adaptive skills, and social skills. Additionally we will measure changes in parent stress and parent satisfaction with autism management. For each of these measures, the parent will be the respondent. Table 1 provides information regarding the measures, format of those measures and respondent.

Table 1. Patient Outcome Assessment Plan – Construct and Format

Measure	Construct	Format	Respondent
Home Situations Questionnaire	Non-compliance Behavior	Questionnaire	Parent
Vineland Adaptive Behavior Scales II	Personal and Social Skills	Interview/ Questionnaire	Parent
Aberrant Behavior Checklist	Problem Behaviors	Questionnaire	Parent
Parenting Stress Index	Parent Stress	Questionnaire	Parent
Parent Satisfaction with Autism Management	Satisfaction	Questionnaire	Parent

Home Situations Questionnaire-Revised (HSQ-R). The original HSQ instrument developed by Barkley measures the presence of child behavior problems across 16 home situations.[61, 62] We will be using a version of this measure that has been adapted for use in children with Autism. This version asks parent to note whether the child has difficulties following rules or expectations in 25 different everyday situations. Questions answered by the parent affirmatively are then rated on a 1 through 9 Likert scale with higher scores indicating more severe noncompliance. Thus the scale yields two scores: a count of "yes" responses (0 to 25) and a severity score (total of 1 through 9 for all items with a "yes" response, for a range of 0 to 225). This total score is usually expressed as the per item mean (total ÷ 25).

Vineland Adaptive Behavior Scales II (VABS). The VABS assess adaptive functioning across several domains: Socialization, Communication, Daily Living Skills, Motor Skills, and an optional maladaptive behavior index.[51] The VABS relies on the parent or primary caretaker to describe what the child actually does in the course of daily living. The scales have been standardized (mean of 100 + 15). Since its reintroduction in 1984,

the VABS has become the most commonly used measure of adaptive functioning in the developmental disabilities field. Children with autism consistently have VABS scores that are considerably lower than their IQ.[63] Indeed, the gap between IQ and adaptive functioning often widens with time in children with autism. In a recent secondary analysis of the Research Units on Pediatric Psychopharmacology (RUPP) Autism Network study of risperidone, age equivalent scores were sensitive to change and easily interpretable.[64] Therefore, the age equivalent scores on the Daily Living Skills scale of the VABS will be used as an outcome measure.

Aberrant Behavior Checklist (ABC). The ABC is a 58-item parent-report measure with five subscales: Irritability (includes agitation, aggression and self-injurious behaviors, 15 items); Social Withdrawal (16 items); Stereotypies (7 items); Hyperactivity (16 items); and Inappropriate Speech (4 items).[65-68] The ABC is commonly used in clinical trials in children with autism and is currently being used in the RUPP and the Studies to Advance Autism Research and Treatment (STAART) studies.

Parenting Stress Index(PSI). The PSI (short form) is a 36-item parent-completed questionnaire for families of children 12 years of age and younger.[69] It has been empirically validated. The PSI has three scales: Parental Distress, Difficult Child Characteristics, and Dysfunctional Parent-Child Interaction. The PSI Short Form is currently used by investigators in the Autism RUPP–PI group.

Parent Satisfaction with Autism Management. We will create a survey to measure parent satisfaction with how their provider handled the management of their child's ASD (Bauer and Swiezy). This survey will be administered at baseline and at 12 months to parents in both the intervention and control groups.

D.3.4 Incentives

For the clinic practices in both study arms, CHICA will be provided free. Other incentives for control practices to participate in the study will include the provision of the "Autism and Pervasive Developmental Disorders Tool Kit" developed by the HANDS in Autism Project Team at the Christian Sarkine Autism Treatment Center located at Riley Hospital for Children in Indianapolis, Indiana. For children and parents in both study arms, a $50 cash incentive will be provided after the completion of each assessment. Assessments will be conducted at 3 month intervals – baseline, 3 months, 6 months, 9 months, and 12 months. Therefore, the total incentive available to a study participant is $250.

D.4 Sample Size (Aims 2 and 3)

D.4.1. Sample Size Calculation for Aim 2

The primary outcome of interest for aim 2 is the proportion of children at the 18 or 24-month visits that will be screened for ASD using the M-CHAT in the intervention group and any ASD specific screening tool in the control group. Dosreis and colleagues found that while 82% of pediatricians reported screening for general developmental delays, only 8% screened for ASD.[3] Though we do not anticipate much variability in this rate at our study clinics, we will use four different rates, 6%, 8%, 10% and 12%, at baseline to estimate intra-clinic correlation. This translates to an intra-class correlation coefficient 0.0041. However, for a conservative estimation of sample size, we will use intra-class correlation 0.0082, twice that of the correlation we estimated.

To be conservative, we will assume 10% of the children are screened for ASD under standard practice in our clinic sites. Based on pilot data involving a different universal screening tool in the pediatric population (i.e., the Ages and Stages Questionnaire), we expect at least 50% of the children at the 18 or 24-month visits will be screened in the intervention clinics. However, we will use a more conservative estimate, that is, 40% of the children will be screened using the M-CHAT.

Using a chi-square test and setting alpha at 0.05, the probability of detecting a statistically significant difference in the proportion of children screened at the 18 or 24-month visits between the intervention and the control group is 90% with an effective sample size of 49 per group. Hence, the total effective sample size (n*) is 98. The unit of randomization is the practice or clinic, but the unit of analysis is the individual patients. Since patients are nested within practice, responses from the patients within practice are likely to be correlated. This intra-cluster correlation must be taken into account in the calculation of sample size and in the analyses. We computed the number of patients per clinic, m, required for the study, using the following equation:

$$m = \frac{1-\rho}{\frac{k}{n^*} - \rho}$$

where ρ = intra-practice correlation, k = number of clinics, mk = total sample size, n^* = total effective sample size = mk/DE, and DE = design effect = 1 + ρ (m-1)[70]

Using 4 clinics and assuming intra-class correlation of 0.0082, we need to recruit 31 children per clinic. We anticipate that drop-out is not a major problem and unlikely to be more than 10% for the primary outcome. We will account for this factor by oversampling patients by 10%. According to our previous experience and data from the four clinics included in this study, at least 1000 patients present to these four clinic sites for 18 or 24-month visits each year, leading to 3000 patients if the study runs for 3 years.

D.4.1. Sample Size Calculation for Aim 3

For aim 3, children between the ages of 18 months through 5 years with newly diagnosed ASD will be included as potential participants. The primary outcome of interest for this aim is the number of activities/involvements (listed in Figure 9) that a child diagnosed with an ASD is receiving. Although better outcomes are expected in the intervention group, no preliminary data are available to support this hypothesis. Hence, the primary purpose of this study is to generate preliminary data on this outcome in order to design a large scale study. Even though the screening rate could be as low as 8% in the intervention group and 40% in the intervention group, the vast majority of children should still receive a diagnosis of ASD by age 5 even without standardized screening at the 18 or 24-month visits. It is likely that we will have at least 80% of the children with a diagnosis of ASD receiving care for that diagnosis by age 5. Though we do not know how many children with newly diagnosed ASD will be available in each of the four clinics, we anticipate more than 20 children in each of the two treatment groups assuming a prevalence of 1 in 150, successful identification of 80% of children with diagnosis ASD, and recruiting about 4000 per group. In 2007, the four study clinics saw 8,528 patients between ages of 0 and 3 years and 3,065 patients between the ages of 4 and 5. Hence, we do not anticipate any problem in having 4000 children per group for this study. We base the sample size justification on estimation and not hypothesis testing. A sample size of 20 per group can be considered adequate for a pilot study given that a sample size as low as 12 in each group provides adequate power for precision in estimating the difference between means in clinical trials in the sense that the gains in precision are less pronounced as sample size exceeds 12.[71]

D.5 Data Analysis (Aims 2 and 3)

For each of the two aims, baseline clinical and demographic data will be compared between the intervention and the control groups. Categorical variables will be examined using chi-square tests and continuous measures with Student's t-tests. The statistical analysis of the data will be performed using the SAS.

D.5.1. Data Analysis for Aim 2

The primary variable for aim 2 is whether a child at the 18 or 24-month visits was screened for ASD using the M-CHAT in the intervention group and any ASD specific screening tool in the control group. For this dichotomous outcome, we will use a non-linear mixed-effects model and will adjust for potential confounders (fixed effects) and clinic effects (random effects). To be specific, if Y_{ik} is a binary variable indicating whether the kth patient in the ith clinic was screened, we have

$$\operatorname{logit}(Y_{ik} = 1) = \alpha_i + \mathbf{X}\boldsymbol{\beta}_{ik} \text{ , where } \alpha_i \sim N\left(0,\ \varepsilon^2\right).$$

The random component α_i correlates outcomes of patients cared by the same clinic. Physicians would not be considered as a nesting factor since the same patients at different visits may not be seen by the same physician. The vector X_{ik} represents fixed covariates and β represents the corresponding coefficients. The fixed covariates will include critical covariates such as treatment group, gender, and other patient and clinic level characteristics. We will also examine potential interaction terms among the covariates, particularly interactions between treatment arm and other covariates. If there is a significant treatment difference, we will report the 95% confidence interval of the treatment difference after adjusting for all confounding factors. The model will be fitted by the SAS procedure NLMIXED.

All secondary dichotomous outcomes, for example, whether a child under the age of 5 undergoes surveillance for ASDs, whether ASD surveillance led to diagnosis of ASD with an ASD specific screening tool and whether a child was referred for comprehensive ASD evaluation will also be analyzed using the same model as described above.

D.5.2. Data Analysis for Aim 3

Aim 3 involves the collection of pilot data and hence we may not have adequate power to detect a difference between the intervention and control groups. We will collect data on the primary outcome, the number of activities/involvements (listed in Figure 9) that a child diagnosed with an ASD is receiving and on secondary multiple patient improvement outcomes at baseline, 6 and 12 months. We will model the longitudinal data on primary outcome measured at baseline, 6 and 12 months and the change in secondary outcomes. For example, change in problem behaviors, parent stress and satisfaction at 6 and 12 months from baseline. The nature of our data is complex, a three-level data structure. The three levels are occasion of measurements, patients and clinics. Patients (level 2) are nested within clinic (level 3) and repeated measurements (level 1) are nested within patient. The model for observation Y_{ijk}, that is, observation for the kth patient at time j and i[th] clinic, is given by

$$Y_{ijk} = \alpha_{0i} + \delta_{0k} + \mathbf{X}'\boldsymbol{\beta} + \varepsilon_{ijk}$$

where the independent components

α_{0i} *distributed as* $N(0, \sigma^2_{(3)})$ δ_{0k} *distributed as* $N(0, \sigma^2_{(2)})$ and

ε_{ijk} *distributed as* $N(0, \sigma^2)$

The random component α_{0i} correlates observations within the same clinic and the random component δ_{0k} correlates repeated observations from the same patient. The vector **X** represents fixed covariates and **β** represents the corresponding coefficients. We will use proc mixed available in SAS to fit this model. The fixed covariates will include treatment group, time, gender, race, age, baseline value of the response variable and other potential clinical outcomes. Baseline value of the primary outcome will not be a covariate in modeling primary outcome since the response vector (Y_{ijk}) will include the baseline outcome. We will first test for the interaction effect between time and treatment group. If there is no interaction effect, we will evaluate the overall treatment difference. Otherwise, treatment difference will be assessed at each time point. If there is a significant difference, we will report the 95% confidence interval after adjusting for all confounding factors.

D.5.3. Missing Data Analysis

We anticipate a low rate of missing data for the primary outcome since our CHICA system showed only 1.3% error rate due to omission of a data field. However, we anticipate a possible dropout rate of 10% for which we have adjusted the sample size. It is very unlikely that dropout will depend on the unobserved outcome. Even if MNAR is true for certain subjects; given the low percentage of missing values, we think the bias induced is negligible. Under circumstances where compromise of power due to missing values is of concern, we will use a multiple imputation procedure to make use of all relevant observed data under the assumption that the data are Missing At Random. The SAS procedure MI and MIANALYZE will be used to implement the procedure.

D.6 Future Work

By the end of this study, we will have adapted CHICA for use in ASD screening, diagnosis and management and fully evaluated its capabilities in improving care in these areas. At that time, we will be uniquely positioned to study the CHICA ASD system in the “real” world, by focusing on widespread dissemination. Dissemination will be facilitated by the open source architecture of the CHICA system. This movement from the “laboratory” of our own clinics to eventual use worldwide is the true goal of translational research, and will complete the long road from development to clinical practice.
