# Supplementary material for: Effect of a Computer-Based Decision Support Intervention on Autism Spectrum Disorder Screening in Pediatric Primary Care Clinics: A Cluster Randomized Clinical Trial
Source: JAMA Netw Open. 2019 Dec 18;2(12):e1917676. doi: 10.1001/jamanetworkopen.2019.17676 (PMC6991212; doi:10.1001/jamanetworkopen.2019.17676)
Supplement: Supplement 2. — Data Sharing Statement [file jamanetwopen-2-e1917676-s002.pdf]

Data Sharing Statement

Downs. Effect of a Computer-Based Decision Support Intervention on Autism Spectrum Disorder Screening in Pediatric Primary Care Clinics. *JAMA Netw Open*. Published December 18, 2019. 10.1001/jamanetworkopen.2019.17676

Data
Data available: No

Additional Information
Explanation for why data not available: Our IRB does not allow for the sharing of the study data.
